# A comparison of recommendations and received treatment for mood and anxiety disorders in a representative national sample

**DOI:** 10.1186/s12888-017-1316-0

**Published:** 2017-05-02

**Authors:** Benjamin A. R. Woolf, Jeanne V. A. Williams, Dina H. Lavorato, Andrew G. M. Bulloch, Scott B. Patten

**Affiliations:** 1 Christ Church, University of Oxford, St Aldate’s, Oxford, OX1 1DP UK; 2 Mathison Centre for Mental Health Research and Education, Hotchkiss Brain Institute, University of Calgary, 4th Floor TRW Bldg., 3280 Hospital Drive NW, Calgary, T2N 4Z6 Canada

**Keywords:** Mental health services/utilization, Anxiety disorders/therapy, Mood disorders/therapy, Alcohol, Counselling/therapy, Street drugs, Smoking, Antidepressant/Medication, Exercise, Canada

## Abstract

**Background:**

The exact nature of treatment and management recommendations made, and received, for mood and anxiety disorders in a community population is unclear. In addition, there is limited evidence on the impact of recommendations on actual receipt of treatment or implementation of management strategies. We aim to describe the frequency with which specific recommendations were made and implemented; and thus assess the size of any gap between the recommendation and implementation of treatments and management strategies.

**Methods:**

We used the Survey ‘Living with a Chronic Condition in Canada - Mood and Anxiety Disorders (SLCDC-MA), a unique crossectional survey of a large (*N* = 3358) and representative sample of Canadians with a diagnosed mood or anxiety disorder, which was conducted by Statistics Canada. The survey collected information on recommendations for medication, counselling, exercise, reduction of alcohol consumption, smoking cessation and reduction of street drug use. We also estimate the frequency that recommendations are made and followed, as well the impact of the prior on the latter. We consulted people with lived experience of the disorders to help interpret our results.

**Results:**

The results generally showed that most people would receive recommendations, almost all for antidepressant medications (94.6%), with lower proportions for the other treatment and management strategies (e.g. 62.1 and 66% for counselling and exercise). Most recommendations were implemented and had an impact on behaviour. The exception to this was smoking reduction/cessation, which was often not recommended or followed through. Other than with medication, at least 20% of the population, for each recommendation, would not have their recommendation implemented. A substantive group also exists who access treatments, and employ various management strategies, without a recommendation.

**Conclusions:**

The results indicate that there is a gap between recommendations made and the implementation of treatments. However, its size varies substantially across treatments.

**Electronic supplementary material:**

The online version of this article (doi:10.1186/s12888-017-1316-0) contains supplementary material, which is available to authorized users.

## Background

Mood and Anxiety disorders are highly common and potentially debilitating disorders. Depression alone affects 350 million people worldwide, is a significant risk factor for suicide, and is among the most burdensome health conditions [1–4]. There has been extensive research on how to treat or manage symptoms of mood and anxiety disorders. For instance, there is evidence showing that pharmacotherapy, [5–7] and specific types of counselling [8–10] are effective treatments. In addition to treatments, other management strategies can also play a role in the reduction of symptoms for mood and anxiety disorders, by changing the lifestyle of those affected. Exercising [11–13] and smoking cessation [14–16] are both effective adjunctive interventions. Alcohol problems are associated, probably causally, with mood or anxiety disorders [17, 18]; a reduction of use is required by many medications [19], and increased use can lead to a worsening of symptoms [20]. Finally, there is evidence that substance use disorders can worsen mood or anxiety disorders [21, 22].

Currently available clinical practice guidelines for major depression are not consistent in differentiating treatments from management strategies. The American Psychiatric Association guidelines [23] and National Institute for Health and Care Excellence guidance [24] emphasize pharmacological and psychological interventions as treatments for depression, but also use the term management. Other guidelines, such as the Royal Australian and New Zealand College of Psychiatrists [25], Canadian “CANMAT” Guidelines [26] and Scottish Intercollegiate Guidelines Network [27] more explicitly refer to strategies such as exercise and smoking cessation as treatments, while also using the term management. In this paper, both terms will be used.

There is limited information about recommendations and treatments received, especially in general population samples is currently available. The evidence that is available is mixed as to the extent to which people are recommended and receive various treatments and implement various management strategies. One recent study, from the United States, found that most participants who met the Patient Health Questionaire-2 screening criteria for major depression did not receive treatment [28]. On the other hand, many studies examining adherence to clinical recommendations find that many people are given recommendations and treated, often in a way compatible with guidelines [29–31]. However, most studies reviewed were conducted using small (e.g. [32]) or not necessarily representative (e.g. [33]) samples and these studies have tended to examine the implementation of guidelines, rather than actual management more generally. Some studies were conducted many years ago and their conclusions may no longer be valid (e.g. [34]).

There is thus little literature assessing the extent to which recommendations are not implemented, after being made. We will call this a recommendation-implementation gap. Anecdotal and some experimental evidence support this construct. For instance, Hetrick et al. [35], in their study of guideline adherence for the treatment of depression in Australia, found that around 25% of those recommended medication did not receive them within 6 weeks.

The objectives of this study are therefore to:

1. To describe the population with diagnosed mood or anxiety disorders.
2. To estimate the frequency with which specific recommendations were made.
3. To describe the treatments actually implemented to treat or manage the disorder(s).
4. To estimate the frequencies with which treatments are implemented given or not given a recommendation, and in doing so assess the size of any recommendation-implementation gap.

This study will examine these with respect to two treatments (medication and professional counselling) and four management strategies (exercise, quitting smoking, reducing alcohol consumption and reducing street drug use).

## Methods

### Study design and procedure

The study used data taken from the Living with a Chronic Condition in Canada, Mood and Anxiety Disorders (SLCDC-MA) survey. The SLCDC is a cross-sectional survey that adopted a sampling strategy linked to a large general population-based health survey, the Canadian Community Health Survey (CCHS) conducted by Statistics Canada [36]. Unlike more typical epidemiological studies, which aim to describe prevalence of disorders [37], the SLCDC surveys are designed to describe the impact of disorders on people’s lives, examining, for instance, the methods used to manage the disorder(s). The sample consisted of respondents to the 2013 Canadian Community Health Survey (CCHS) who reported having a mood or anxiety disorder, and agreed to participate in the SLCDC. Details about the design of the survey, including exclusion and inclusion criteria, can be found at the Statistics Canada website [38].

### Data collection

The data was collected between October 23, 2013 and March 31, 2014 using computer-assisted interviewing. This approach helped to ensure high data quality since the computerized administration helps to ensure that the correct type of answer was provided during survey interviews. The interview was field tested by Statistics Canada in 2013. During the data collection, no proxy interviewing was done. The interviews were conducted by telephone in the respondents preferred language. More detail can be found from the Statistics Canada website [38].

### Population

The target population for the CCHS was the Canadian household population. The SLCDC targeted those, over the age of 18, living in the ten provinces who had a professional diagnosis of a mood or anxiety disorder. This came to approximately *n* = 5875. The survey excluded anyone living on a reserve or other Aboriginal settlement; full-time members of the Canadian Forces; the institutionalized population and residents of certain remote regions [38]. In total this represented approximately 3% of the target population. Statistics Canada estimates that there was a 69% response rate, based on consideration both of household and individual responses. We excluded 3 respondents for not being over 18 when the SLCDC was conducted. This resulted in a final sample size of 3358.

### Measures

In order to establish what recommendations had been made, all participants were asked, ‘Has a doctor or other health professional ever suggested [treatment/management strategy] for your mood or anxiety disorder?’ for the following: medication, counselling, exercise, reduction of street drugs, reduction of alcohol use, or quitting smoking.

For exercise, quitting smoking, stop using street drugs and reducing alcohol, participants were asked ‘Did you ever [implement recommendation] to help manage your mood or anxiety disorder?’. If they met the restrictions (described in Additional file 1), participants were then asked ‘Are you still [following the recommendation]?’.

For medication use all participants were asked ‘Currently, are you taking any prescription medications for your mood or anxiety disorder?’ Those who did not say yes, were then asked ‘Have you ever taken any prescription medications for your mood or anxiety disorder?’

For counselling, participants who reported having seen a mental health professional in the past 12 months were asked, ‘In the past 12 months, did you receive psychological counselling to help manage your mood or anxiety disorder?’.

The other measures used and the restrictions applied are described in Additional file 1.

### Statistical analysis

Univariate analysis was used to establish the estimated weighted proportions and associated confidence intervals. STATA (version 14) was used to conduct the analysis [39].

To establish the conditional frequencies of having done a treatment or implemented a management strategy given a recommendation, the ‘done in past 12 months’ and ‘ever done’ variables were cross tabulated with the respective recommendation variables.

In order to make the variables comparable, some were restricted. Estimates of ‘Street Drug’, ‘Alcohol’ and ‘Smoking’ variables were restricted to respondents who did not express having stopped using or never using the respective substance since being diagnosed (leaving 13.9%, 16.6%, and 43.2% of the sample respectively). The ‘done therapy in past 12 months’ variable was originally restricted to those who reported seeing a professional in the past 12 months (leaving 76.9% of the total sample). However, because this is a pre-condition of being able to receive professional counselling, this variable was re-coded so that it has the same (unrestricted) universe as the recommendation variable. In addition, the sample of follow up questions was also expanded so that they would be comparable with the prior question(s).

As recommended by Statistics Canada, 500 replicate bootstrap weights were applied to ensure the accuracy of the standard errors and to account for the complex sampling (cite user guide). These weights also contain post-stratification demographic adjustments and adjustment for non-response. All data analysis was conducted in a Statistics Canada Regional Data Centre using STATA 14 [39].

### Patient group

To expand the SLCDC’s aim of assessing the experience and impact of the disorder on the everyday lives of the sufferers, we elicited the help of a patient group in interpreting the data. These were members of the Organization for Bipolar Affective Disorder. As they have lived experience of the disorder, they have knowledge not necessarily accessible to a researcher. This should increase the validity of the interpretations drawn from the results. The group consisted of 12 people, who self-identified as having a mood or anxiety disorder, most of whom had been diagnosed and treated. The results were presented and explained to them by a slide show followed by a discussion session in a community centre in central Calgary. It is important to emphasize that this component of the study was not undertaken as a qualitative study, but rather as a consultation in keeping with the principles and values of public engagement in research [40].

## Results^1^

### Socio-demographic factors

**Fig. 1:**
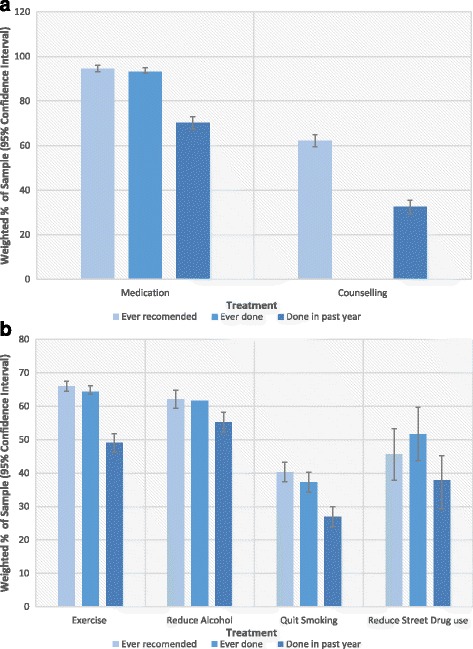
**a** Percentages of Treatments Received and Recommended (error bars are 95% CIs). **b** Percentages of Management Strategies Implemented and Recommended (error bars are 95% CIs)

Table 1 shows that the population is mostly made of relatively wealthy (60.9%, CI 58.4-63.5%, have a household income over $50,000 CAD/year), working (53.0%, CI 50.0-55.9%, worked in previous week), middle aged (mode age 45-54 years), heterosexual (92.3%, CI 90.0-94.6%) female (63.6% CI 61.6 – 65.7%) living in urban environments (82.3%, CI 80.4-84.2%) (Table 2). Around 23% (CI 20.1-25.3%) had not seen any health professional in the past 12 months. Among respondents 45.3% (CI 42.3-48.21%) reported having only a mood disorder, 30.7% (CI 28.1-33.4%) reported having a mood and anxiety disorder and 24.0% (21.4-26.5%) reported only an anxiety disorder (Fig. 1).

**Table 1:** Characteristics of the participants (*N* = 3358)

Variable	Level	Frequency (%)	95% CI
Age	18-24	9.1	7.6 - 10.7
	25-34	16.8	14.6 - 19.0
	35-44	18.3	16.2 - 20.4
	45-54	20.9	18.2 - 23.5
	55-64	19.9	18 .0 - 21.9
	65-74	11.0	9.8 - 12.1
	74+	4.0	3.3 - 4.8
Sex	Female	63.6	61.6 – 65.7
	Male	36.4	34.3 – 38.4
Disorder type	Mood only	45.3	42.3 - 48.21
	Anxiety only	24.0	21.4 – 26.5
	Mood & anxiety	30.7	28.1 – 33.4
Sexuality	Heterosexual	92.3	90.0 - 94.6
	Homosexual	4.2	2.3 - 6.2
	Bisexual	3.5	2.2 - 4.8
Income	I > 50 k/yr	60.9	58.4 - 63.5
	I < 50 K/yr	39.1	36.5 – 41.6
Marital status	Married	43.7	40.8 - 46.5
	Common-law	12.5	10.4 - 14.5
	Widowed	4.1	3.3 - 4.8
	Separated	4.9	3.7 – 6.2
	Divorced	8.7	7.2 - 10.2
	Single	26.1	23.7 - 28.6
Work status	Job in past week	53.0	50.0 - 55.9
	Job, but absent, in past week	7.7	6.0 - 9.4
	No job in past week	30.0	27.5 - 32.6
	Unable to work	9.3	7.7 - 10.8
House hold education	Not graduated secondary school	6.1	5.0 - 7.1
	Graduated secondary school	14.8	12.7 - 16.8
	Some post-secondary	4.0	3.0 - 5.2
	Graduated post-secondary	75.1	72.7 - 77.4
Immigrant status	Immigrant	12.5	9.9 - 15.1
	Non-immigrant	87.5	84.9 - 90.1
Habitat	Urban	82.3	80.4 - 84.2
	Rural	17.7	15.8 - 19.6
Health professionals seen in past 12 months	None	23.0	20.1 - 25.3
	Social worker/counsellor	17.9	15.8 - 20.1
	Psychologist	16.9	14.2 - 19.5
	Psychiatrist	19.7	17.3 - 22.0
	Family doctor	65.0	62.2 - 67.8
	Nurse	9.7	07.9 - 11.5
	MD other	5.2	04 - 06.4
	Health professional, other	2.2	01.4 - 03.0

**Table 2:** frequency of use of treatment or management strategy

Variable	Level	Frequency (%)	95% CI
Number of counselling sessions	1 or 2	19.7	14.1 – 25.4
	3-5	25.2	20.0 – 30.4
	6-9	17.7	13.5 – 21.8
	10 or more	37.4	31.4 – 43.3
Frequency of exercise	Every day	25.3	21.5 – 29.1
	4-6 times per week	22.4	19.1 – 25.8
	2-3 times per week	41.1	36.5 – 45.7
	1 or fewer times per week	11.2	08.6 – 13.8
Number of regular medication	0	03.1	02.1 – 04.0
	1	65.8	62.4 – 69.3
	2	18.4	15.6 - 21.2
	3 or more	12.7	10.3 – 15.1
Number of irregular medication	0	72.5	69.6 – 75.5
	1	22.6	19.7 – 25.4
	2	03.2	02.0 – 04.4
	3 or more	01.7	00.9 – 02.5

### Medication

Medication was recommended to 94.6% (CI 93.1-96.1%) of the population. A similar proportion had “ever done” (93.2%, CI 91.5 – 94.9%) treatment with a medication, but fewer had done it in the past 12 months (70.2% 67.4 – 73%). Although still high, this suggests that medication treatment is time-limited for a sizable proportion of those treated in the population. There is insufficient data to assess the extent to which the difference between ever and past 12-month use is due to successful treatment outcomes followed by discontinuation, discontinuation due to non-response, or non-adherence.

**Fig. 2:**
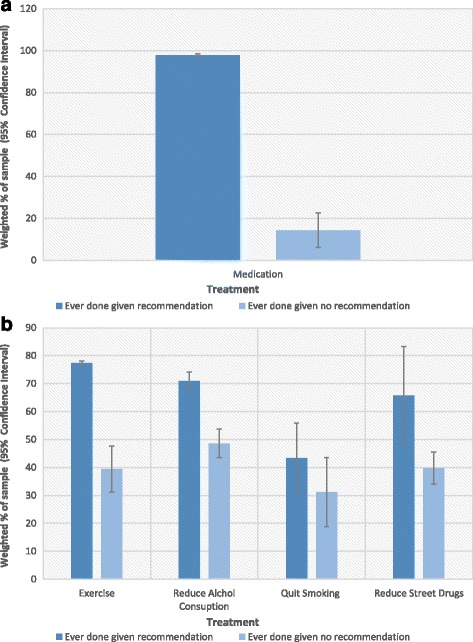
**a** Comparison of Ever Doing Medication given Recommendation or no-Recommendation (error bars are 95% CIs). **b** Comparison of Ever Implementing Management Strategies given Recommendation or no-Recommendation (error bars are 95% CIs)

**Fig. 3:**
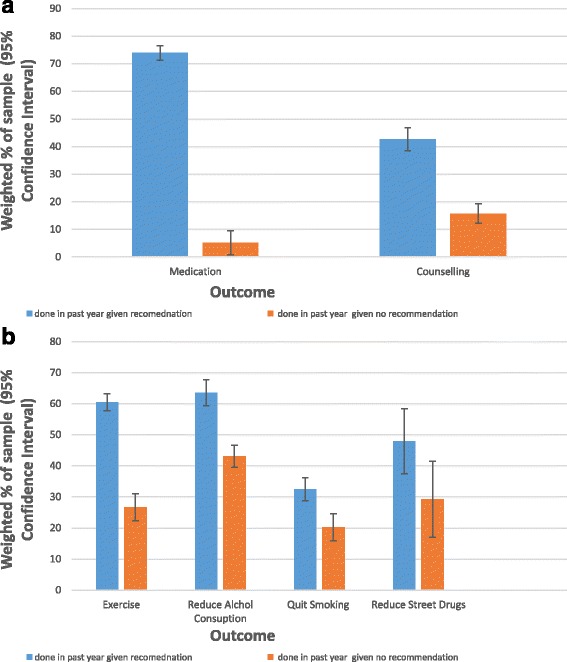
**a** Comparison of Treatments done past 12 months given recommendation or no-recommendation (error bars are 95% CIs). **b** Comparison of Management Strategies Implemented in the past 12 months given recommendation or no-recommendation (error bars are 95% CIs)

Both Fig. 2a and Fig. 3a show a difference between those who have ever taken medication, or taken it in the past 12 month, given a recommendation (97.7%, CI 96.9 – 98.5%, and 73.9%, CI 71.2 – 76.6% respectively) relative to the same frequencies when there was no recommendation (14.3%, CI 6.1 – 22.6%, and 5.2%, CI 0.8 – 9.6%, respectively). Thus a significant proportion of respondents report taking a medication even though it had not been recommended by a health professional.

This is consistent with the patient group’s experience, virtually all of whom took medication. However, they knew people who had not followed this recommendation due to side effects and stigma.

### Counselling

62.1% (CI 59.4-64.8%) were recommended counselling. However, only 32.5% (CI 29.5 – 35.5%) reported having taken counselling in the past 12 months (ever participating in counselling was not assessed). More than half of those who received counselling reported receiving more than six sessions, with 37.4% (CI 31.4 – 43.3%) getting over ten. This implies they received a clinically significant number of sessions [41], although, as we cannot establish which type of therapy each participant received, this can only be an estimate.

Figure 3a shows a difference between those who have taken counselling in the past 12 months with a recommendation (42.7%, CI 38.5 – 46.5%) or no recommendation (15.7% CI 13.5 – 19.2). This indicates that recommendations do affect behaviour. Surprisingly, as psychotherapy is often thought to be under implemented, about a quarter of users do not have a recommendation.

These results also did not surprise the patient group. As therapy in Canada is expensive, people in their experience would not use professional counselling indefinitely. In addition, they knew of people who had sought counselling as a means of psychological support, even when they were not recommended to do so.

### Exercise

Exercise was recommended to 66.0% (CI 63.1-68.9%) of the population. A similar proportion had ever employed (64.4%, CI 61.4 – 67.3%) exercise as a means of managing symptoms, but fewer had continued to do so in the past 12 months (49.0%, CI 45.9 – 52.0%).

Both Fig. 2b and Fig. 3b show a difference between those who have ever done exercise, or employed it in the past 12 month, given a recommendation (77.3%, CI 74.1 – 80.5%, and 60.5%, CI 56.8-64.2%, respectively) or no recommendation (39.4%, CI 34.4 – 44.5%, and 26.7%, CI 22.4 – 31.0%, respectively). Thus, having received a recommendation was associated with greater implementation and adherence. Even though health professionals recommended exercise less often than medications or therapy, many patients implemented exercise anyway.

The patient group indicated that personal experience guides this type of action and many reported discovering on their own that exercise improves mood. In addition, they thought that factors like lacking structure in one’s life and the stage of the disorder (whether one is still learning how to cope with the disorder) were important factors in inhibiting exercise, irrespective of a recommendation.

### Reduce alcohol consumption

Alcohol reduction was recommended to 62.1% (CI 54.4 – 69.8%) of the population who drank over 10 to 15 drinks per week post diagnosis. A similar proportion of this group had ever done (61.6%, CI 53.6 – 69.6%) alcohol reduction, or done it in the past 12 months (55.2%, CI 46.8 – 63.6%). Given the restricted sample, this implies a low frequency of recommendation compared to the previous treatments.

Neither Fig. 2b nor Fig. 3b show a difference in the proportion of participants who had ever reduced alcohol, or done it in the past 12 months, based on if they get a recommendation (70.9, CI 60.9 – 80.9, and 63.6%, CI 53.1 – 74.1%, respectively) or not (48.6, CI 36.2 – 61.1%, and 43.%,1 CI 30.8 – 55.4%, respectively). However, the high proportion of respondents who implemented alcohol reduction without a recommendation might account for this. There is also a high retention rate (perseverance in implementing this strategy in the past year relative to having ever done so), irrespective of a recommendation.

This is consistent with the experience of the patient group. Many reported that they had stopped independently of a recommendation because they found that alcohol interfered with their medication. A minority told us that they continued to drink as a way of escaping from the day-to-day strain of living with a mood or anxiety disorder.

### Quitting smoking

Quitting smoking was recommended to 40.3% (CI 35.6-45.0) of the population who had smoked post diagnosis. A similar proportion had ever done (37.2%, CI 32.7 – 41.7%) it, but fewer continued to do so in the past 12 months (26.9 CI 22.6 – 31.2%). Even though quitting is associated with improved mental health, it does not seem to be a common recommendation or consistently implemented.

Neither Fig. 2b nor Fig. 3b show a difference in the proportion of the population who had ever quit smoking, or done in the past 12 months, based on if they get a recommendation (43.4%, CI 35.8 – 51.0%, and 32.5%, CI 25.0-40.0%, respectively) or not (31.2%, CI 25.4-36.9%, and 20.2%, CI 15.2 – 25.1%, respectively). This suggests that recommendations do not strongly impact people’s behaviour. However, they do indicate relatively high retention among those who do stop smoking, irrespective of a recommendation.

The low proportion of smoking cessation was contrary to the experience of the patient group, most of whom had stopped. However, like with alcohol, they knew many people who continued to smoke as a means of escape. Of those who had quit, some had done so because of recommendations while other had done so because it interfered with medication.

### Reduce street drug use

Reduction of street drug use was recommended to 45.6% (CI 36.6-54.5%) of the population who had used street drugs post diagnosis. A similar proportion had ever reduced street drug use (51.7%, CI 42.7 – 60.6%) or done the treatment in the past 12 months (37.8%, CI 29.3 – 46.2%).

Figure 2b shows a difference between those who have ever reduced street drug use given a recommendation (65.8%, CI 52.5 – 79.1%) or no recommendation (47.9, CI 34.1 – 61.6). However, Fig. 3b does not decisively show a difference between those who have reduced street drug use in the past 12 months given a recommendation (39.8%, CI 28.8 – 50.7%) or no recommendation (29.3%, CI 19.4 – 39.0%). This indicates that there is a higher retention rate among those who reduce street drug use without a recommendation than with one.

Most member of the patient group had stopped using street drugs. Many felt that this had improved their condition or made their drugs more effective. But, they were not surprised by the low levels of recommendation or implementation: in their experience people used them as a means of escape from living with the disorder and some doctors would not tell people to stop using them for that reason (or would be unaware of its use).

## Discussion

### Socio-demographics

The socio-demographics are generally consistent with expectations. The low proportion of immigrants is consistent with the ‘healthy immigrant effect’ [42, 43]. The 2:1 female: male ratio is well established for this population [44–46]. The average age is older than traditionally thought to be [4]. However, Olfson et al. (2016) found a similar result [28]. The proportion of heterosexuals is consistent (given a national prevalence of 97% [47]) with previous findings that non-heterosexuals are at risk of mood or anxiety disorders [48]. The unemployment rate (around half) is high relative to the national average (6.7% in 2014) [49].

### Absence of recommendations

As the recommendations reviewed in this paper are often viewed as effective treatments for mood or anxiety disorders [50], one might expect a high proportions of recommendations would be made. The low frequency of recommendations made is thus worrying. With the exception of medication, about 35-60% of the relevant population would not receive the various specific recommendations.

The absence of a recommendation is not necessarily a problem. Doctors use their judgment in making recommendations, and not all treatments are appropriate for every person. For instance, someone suffering from severe major depressive disorder might lack the motivation to be able to exercise.

Even allowing for this, the relative lack of recommendations seems too great. Smoking, for instance, is known to be a major cause of physical problems and also impacts on the severity and prognosis of a mood or anxiety disorder [14]. Yet, under half of those who smoked post diagnosis had been recommended to stop.

These results may point towards a narrow perspective on treatment in Canada, emphasizing medications, but less often recommending other approaches.

### Impact of a recommendation

One reassuring finding is that recommendations do appear to have an impact on behaviour. Nearly every recommendation was associated with an increased implementation of the treatment. Often this was true for both the ‘ever done’ and ‘done in past 12 months’ variables, implying that recommendations can have a lasting impact.

The two exceptions to this trend are alcohol reduction and smoking. The result in Figs. 2b and 3b, indicate that, in the case of alcohol reduction, this is due to people adopting the strategy without a recommendation. This interpretation is not applicable to smoking. Only around 40% of those who received the recommendation to quit smoking had ever done so. This indicates that people do not follow recommendations to quit smoking. They may have tried to quit, but been unsuccessful.

### Recommendation-implementation gap

A goal of this analysis was to help quantify a recommendation-implementation gap. As no recommendation or management strategies was fully implemented, the findings support a treatment-implementation gap. However, the nature of the gap varies for each recommendation and management strategies.

Medication, for instance, was taken by the vast majority of those who were recommended to take it. Although there was a very small group (under 3%) who did not follow this recommendation. Likewise, exercise seemed widely implemented.

The gap seems more prevalent for the other management strategies, especially smoking cessation, which was ever implemented in under 50% of those to whom it was recommended (and it was only recommended to a minority of smokers). The possible use of these (alcohol, street drugs and tobacco) as a means of escaping the daily strains of living with a mood or anxiety disorder implies that finding an alternative means of relaxation may be important, e.g. mindfulness-based medication strategies or relaxation techniques.

The existence and nature of a gap is hard to assess with respect to counselling, due to the lack of an ‘ever done’ variable. On the one hand, a large proportion of those who were recommended to do it had not done so in the past 12 months. On the other, evidence based psychotherapies for mood and anxiety disorders are time limited and most of those who had done it in the past 12 months seemed to have done (or be on route to completing) an adequate number of sessions.

The finding of this gap has important implications for the wider literature and guiding public health policy. There is extensive research on the construct of a treatment gap, defined as a difference in the prevalence of a disorder in the population and the prevalence of treatment [51–53]. The recommendation-implementation gap gives an indication how much of the treatment gap is due to people not seeking help or getting a recommendation versus a failure of the health system to implement recommendations. Our results show that, on the whole, a small part of the treatment gap is due to a recommendation-implementation gap. The lack of recommendations would appear to be a much greater problem. However, as the sample is only of those who have seen a professional, we cannot assess the general construct of a treatment gap.

### Treatment without recommendations

Figures 2 and 3 imply that a substantial minority of respondents receive treatments or management strategies without recommendations. This is strikingly the case with medication, 14.3% of those who have ever taken medication did so without a recommendation. Yet, one would not expect medications to be available to those without prescriptions. However, the people with lived experience who were consulted in this project were less surprised. They were also aware of ways to get medication without a recommendation, for instance using a friend or relative’s prescription.

### Generalisability

These results should be highly generalisable to the Canadian household population. As the sample frame is based on a randomised survey design for the general population, and the bootstrapping means that the estimates should be valid for the target population.

### Limitations

The study has several limitations. As a cross-sectional study, one cannot draw causal conclusions from the results, since temporality cannot be confirmed. We were unable to evaluate the quality or appropriateness of the treatments received by, and recommendations made to, respondents. Not all possible treatments were covered, e.g. neurostimulation treatments (e.g. Electroconvulsive therapy, or Repetitive transcranial magnetic stimulation) and details were lacking, for example concerning specific medications recommended, and their prescribed dosages. The sample was limited to those who had sought professional help or advice. This makes it little use in assessing the care received outside this population, e.g. those prevented by stigma. Future studies should explore these issues. Ideally, a diagnostic interview would have confirmed the self-reported professional diagnoses. A larger sample size might have resulted in greater precision and allowed more stratification. Finally, the survey relied extensively on retrospective- and self-report (e.g. ever taken a medication for treatment), creating a risk of recall bias.

## Conclusion

Overall, the results paint a complex picture, but with some common themes. Recommendations tend to impact people’s behaviour and be implemented. However, treatments are often not recommended enough. Other than medication, nearly all of the treatments or management strategies were recommended to under 65% of the relevant population. A substantive minority (at least 20% for nearly all treatments or management strategies) do not have their recommendations implemented. This is evidence for a recommendation-implementation gap. However, there is large variation in its strength across treatments. The results indicate that many people access treatments or management strategies without recommendations. Management strategies can be an effective means of improving outcomes in these disorders. Our results imply that people might have trouble following them. The smoking results were the most worrying, showing that too few recommendations were made, or were not followed through if made. Future studies should look beyond the concept of a treatment gap to examine how best to implement treatments and increase the frequency that recommendations are made.

## Additional file

Additional file 1: Full description of questions asked to respondents, and restrictions applied to the answers. (DOCX 12 kb)

## Abbreviations

CANMAT: Canadian network for mood and anxiety treatments
CCHS: Canadian community health survey
SLCDC-MA: Living with a chronic condition in canada - mood and anxiety disorders

## Footnotes

1 Statistics Canada rules prescribe releasing only weighted frequencies. None of the results given are thus indicative of the raw number of respondents.
