# Supplementary material for: A comparison of recommendations and received treatment for mood and anxiety disorders in a representative national sample
Source: BMC Psychiatry. 2017 May 2;17:155. doi: 10.1186/s12888-017-1316-0 (PMC5414207; doi:10.1186/s12888-017-1316-0)
Supplement: Additional file 1: — Full description of questions asked to respondents, and restrictions applied to the answers. (DOCX 12 kb) [file 12888_2017_1316_MOESM1_ESM.docx]

Appendix 1: questions asked to respondents.

For some treatments, participants were asked for the frequency of use. Those who had done counselling in the past 12 months were asked ‘In the past 12 months, how many psychological counselling sessions did you have?’, those who had taken medication in the past 12 months were asked ‘Currently, how many different types of prescription medications do you take on a regular basis for your [mood disorder/anxiety disorder/mood disorder and anxiety disorder]?’ and ‘Excluding medications taken on a regular basis, currently, how many different types of prescription medications do you take as needed for your [mood disorder/anxiety disorder/mood disorder and anxiety disorder]?’, those who had not reported not currently doing exercise because of diagnosis were then asked ‘How often do you do this?’.

Socio-demoFigureic variables include age, sex, household education (less than secondary school graduation, secondary school graduation, some post-secondary, and post-secondary graduation), medication insurance (yes or no), household income (above or below 50,000 CAD per year), sexuality (heterosexual, homosexual, bisexual), employment status in the past week (employed and worked in past week, employed but did not work in past week, unemployed, cannot work), marital status (married, common law, divorced, single, separated), habitat (urban, rural) and immigrant status (immigrant or non-immigrant).

To establish who participants had seen, they were asked ‘During the past 12 months, have you seen, or talked on the telephone to, any of the following people about your mood or anxiety disorder?’. They could choose from: ‘family doctor or general practitioner’, ‘psychiatrist’, ‘psychologist’, ‘social worker, counsellor, or psychotherapist’, ‘nurse or nurse practitioner’, ‘social worker, counsellor, or psychotherapist’, ‘other medical doctor or specialist ‘, ‘other health professional’, or ‘none’.

To establish which disorder(s) participants have, they were asked ‘To begin, do you have a mood disorder such as depression, bipolar disorder, mania or dysthymia that has been diagnosed by a health professional’. Those who responded ‘no’ to this where then asked if they ever had a mood disorder. If they responded in the affirmative, they were asked ‘What kind of mood disorder [do/did] you have?’, with the options of depression, seasonal affective disorder, dysthymia, bipolar or mania, or other to choose from. Participants were also asked ‘Do you have an anxiety disorder such as a phobia, obsessive-compulsive disorder or panic disorder that has been diagnosed by a health professional?’ with an equitant follow up question if they responded with ‘no’. They were then asked ‘what kind of anxiety disorder [do/did] you have?’, with the options of phobia, social phobia, agoraphobia, obsessive compulsive disorder, generalised anxiety disorder, panic disorder, post-traumatic stress disorder, or other to choose from.

Many of the questions were asked to different sub populations. All recommendation variables were asked to all participants. The exception to this was the street drug variable, which was asked only to those who reported having used them in the past 12 months. The ‘ever done treatment’ variables were then asked to a restricted sub population (described in the analyses section). The exceptions to this are medication and exercise which were asked to all participants. The ‘followed in the past 12 months’ variable was asked to all those who said that they had ever done the treatment. The exception to this is medication, where the ‘followed in the past 12 months’ was asked first and the ‘ever done’ as a follow up for those who said no.
